# Fatal outcome related to drug reaction with eosinophilia and systemic symptoms: a disproportionality analysis of FAERS database and a systematic review of cases

**DOI:** 10.3389/fimmu.2024.1490334

**Published:** 2024-12-16

**Authors:** Chunsu Liang, Pengjiao An, Yizhou Zhang, Xin Liu, Bo Zhang

**Affiliations:** ^1^ Department of Pharmacy, Peking Union Medical College Hospital, Chinese Academy of Medical Sciences and Peking Union Medical College, Beijing, China; ^2^ State Key Laboratory of Complex Severe and Rare Diseases, Peking Union Medical College Hospital, Beijing, China

**Keywords:** drug reaction with eosinophilia and systemic symptoms, drug-induced hypersensitivity syndrome, FDA adverse event reporting system, fatal cases, drug adverse reaction, systematic review, disproportionality analysis

## Abstract

**Background:**

Drug rash with eosinophilia and systemic symptoms (DRESS) is a life-threatening severe cutaneous adverse reaction.

**Objective:**

This study aims to study fatal DRESS cases using FAERS database and systematic review.

**Methods:**

Data of the FDA Adverse Event Reporting System (FAERS) database were extracted and manipulated. Articles from Pubmed, Embase and CINAHL databases were screened.

**Results:**

0.13% of the adverse events submitted to FAERS was identified as DRESS and the percentage of fatal cases was up to 6.62%. The top five drugs calculated to induce DRESS with the highest number of reported cases were allopurinol, lamotrigine, vancomycin, amoxicillin and carbamazepine. The top five drugs statistically related to fatal outcome with the highest number of reported cases were allopurinol, vancomycin, trimethoprim, sulfamethoxazole and lamotrigine. Skin manifestations remained the main reason for admission and the average time from dose to rash onset was 27.19 days. The most commonly cited culprit medication type were antibiotics (50.00%), anti-gout agents (15.38%) and anti-epileptic drug (11.54%).

**Conclusions:**

We discussed fatal cases of DRESS through FAERS system and case reports, hoping to raise awareness when using relevant drugs.

## 1 Introduction

Drug-induced hypersensitivity syndrome (DIHS), also referred to as drug reaction with eosinophilia and systemic symptoms (DRESS), is a severe cutaneous adverse reaction (SCAR) characterized by the presence of a rash, fever, and involvement of hematologic and visceral organs. The etiology of DRESS syndrome is currently not well-defined. Proposed mechanisms include aberrations in drug detoxification enzymes leading to the buildup of reactive drug metabolites, sequential reactivation of herpesviruses, such as cytomegalovirus, Epsteine-Barr virus (EBV), human herpesvirus (HHV) -6 and -7, and genetic predisposition linked to specific human leukocyte antigen alleles. However, a comprehensive understanding of the pathogenesis of DRESS syndrome is still lacking (1).

DIHS/DRESS is a highly debilitating and potentially life-threatening condition, historically associated with a mortality rate estimated at 10%, while recent studies have indicated mortality rates ranging from 1.2% to 6.1% (2). In the pediatric population, systematic reviews have reported mortality rates ranging from 3% to 5.4% (2).

To our best knowledge, so far there has been no article summarizing reports of death cases of DRESS. Therefore, we analyzed the signals of DRESS in FDA Adverse Event Reporting System (FAERS) and made a systematic review of the death case reports on DRESS, in order to provide reference for the security of drug usage in clinical practice.

## 2 Methods

### 2.1 Analysis of FAERS database

The FAERS system serves as a spontaneous adverse event reporting mechanism, gathering reports from diverse sources such as healthcare professionals, patients, and manufacturers on a global scale. Its significance lies in its ability to identify safety concerns promptly, particularly pertaining to newly introduced medications and infrequent adverse events. OpenVigil 2, a pioneering web-based tool for pharmacovigilance analysis, utilizes the open FDA online interface provided by the FDA to access pharmacovigilance data from FAERS in the United States. Our study employed OpenVigil 2 to extract the FAERS data from 2003Q4 to 2023Q3. “Drug reaction with eosinophilia and systemic symptoms” was searched as preferred term (PT) for targeted adverse events. Arbitrary drug names (generic names, brand names, abbreviations, and so on) was mapped to unique drug names by using Pubchem and Drugs@FDA. We attempted to identify same active ingredient in different drugs according to the WHO Anatomical Therapeutic Chemical (ATC) classification.

We did the disproportionality analyses through OpenVigil 2 and Microsoft^®^ Excel 2016. Proportional reporting ratio (PRR) and reporting odds ratio (ROR) was calculated to assess the association between drug and events. Higher PRR or ROR suggests stronger association. Roughly, PRR/ROR values greater than 2 indicate that this drug-adverse event-combination is 2-fold more likely than all other combinations. Adverse events reported to FAERS database will be considered as positive signals if fulfilled the criteria: (1) PRR ≥2, χ2 ≥4 and ≥3 case; and (2) reporting odds ratio (ROR)>1; and (3) 95% confidence interval (CI) of ROR>1.

### 2.2 Systematic review

#### 2.2.1 Search strategy

A comprehensive literature search was conducted in Pubmed, Embase, and CINAHL databases, encompassing articles published on or before August 2023. The objective was to identify case reports and case series of deaths pertaining to adverse drug reactions (ADR) associated with DRESS. Additionally, the reference lists of the identified articles were scrutinized. The search strategy employed a combination of search terms including ‘drug reaction with eosinophilia and systemic symptoms’, ‘DRESS’, ‘drug-induced hypersensitivity syndrome’, ‘DIHS’, ‘case report’, ‘case reports’, ‘case series’, ‘case study’, ‘case studies’ and other related terms, connected using logical operators ‘AND’ and ‘OR.’ Both MeSH and text-word searching methods were utilized in the search process.

#### 2.2.2 Selection criteria

The selection process involves the inclusion of studies that satisfy the subsequent criteria: (1) the publication type is either a case report or case series of human, (2) the studies describe cases involving mortality subsequent to the dress diagnosis, and (3) the studies provide comprehensive information regarding patients and their adverse reactions. Conversely, studies that cannot conform to the aforementioned study type, uncertain to be caused by other drugs, constitute secondary literature, or lack access to full-text or essential information are excluded. According to the RegiSCAR score, DRESS can be categorized into four levels: no case (score < 2), possible case (score 2~3), probable case (score 4~5), and definitive case (score > 5) (3). For our study, we specifically focused on probable and definitive DRESS cases, which were identified based on a RegiSCAR score of 4 and above. Additionally, The selection of publications is conducted autonomously by two reviewers, with any discrepancies being resolved through consensus.

#### 2.2.3 Data extraction

The extracted information encompasses various aspects, namely: (1) personal details of the patient such as age, gender, past medical history, time and place of death, and cause of death; (2) medication-related information including the specific drug responsible for DRESS, the commencement and cessation dates of medication administration; and (3) pertinent details pertaining to DRESS, such as diagnostic test indicators, affected organs or systems, and the Regi S-CAR scores.

## 3 Results

### 3.1 DRESS in FAERS

From 2003 Q4 to 2023 Q3, 0.13% of adverse events submitted to FAERS (15,751 of 11,737,133) was identified as DRESS. The percentage of fatal cases was up to 6.62% (1,043 of 15,751). Among all DRESS patients, there were slightly more females than males (53.9% vs 46.1%), with an age of 47.5 ± 22.4 years Among all DRESS patients who died as a result, there were slightly more females than males (53.3% vs 46.1%), with an age of 54.3 ± 24.4 years. There was no significant difference in the gender ratio between the deceased patients and all patients (p>0.01), but there was a statistical difference in age (p<0.001). During the period evaluated, 2324 different drugs were considered as suspected drugs, while 618 of them was positive signals, namely statistically related to induce DRESS, and 191 of them was statistically related to death. The top five drugs calculated to induce DRESS with the highest number of reported cases were allopurinol (n=2375), lamotrigine (n=1732), vancomycin (n=1564), amoxicillin (n=1175) and carbamazepine (n=1163) (**Table 1**).

**Table 1:** Association analysis of DRESS and suspected drugs, sorted by the number of reported cases.

Drugs	Cases (n_1_)	PRR	χ^2^	Drugs with fatal outcome	Deaths (n_2_)	PRR	χ^2^
Allopurinol	2375	35.04	66753.98	Allopurinol	280	50.19	9847.91
Lamotrigine	1732	19.30	26762.54	Vancomycin	104	27.91	2407.23
Vancomycin	1564	47.95	64803.64	Trimethoprim	87	12.80	857.66
Amoxicillin	1175	15.58	14846.29	Sulfamethoxazole	84	13.33	869.99
Carbamazepine	1163	24.44	24222.75	lamotrigine	84	15.89	1064.69
Trimethoprim	1067	14.25	12262.20	Minocycline	74	126.80	8471.92
Sulfamethoxazole	1040	15.09	12779.84	Phenytoin	68	30.44	1784.42
Rifampicin	1038	54.46	50904.99	Levetiracetam	68	14.48	785.87
Levetiracetam	797	7.28	4098.65	Furosemide	63	3.10	82.23
Valproic acid	760	7.35	3969.42	Rifampicin	62	38.74	2110.72
Phenytoin	753	23.04	15115.38	Ceftriaxone	58	23.23	1145.17
Ceftriaxone	697	26.96	16650.70	Carbamazepine	55	18.96	870.07
Piperacillin	650	31.62	18473.30	Pantoprazole	52	4.28	121.11
Isoniazid	635	44.70	26030.60	Amoxicillin	48	13.93	537.83
Tazobactam	629	30.24	17069.80	Methylprednisolone	48	4.95	140.65
Furosemide	571	2.96	710.62	Prednisolone	44	3.40	69.09
Pyrazinamide	553	69.42	35962.90	Omeprazole	43	3.11	57.07
Ethambutol	550	54.98	28116.87	Phenobarbital	42	41.76	1566.11
Ciprofloxacin	527	8.11	3169.73	Isoniazid	37	29.39	952.50
Levofloxacin	524	6.29	2252.83	Saccharated iron oxide	36	23.64	732.42

The top five drugs statistically related to fatal outcome with the highest number of reported cases was allopurinol, vancomycin, trimethoprim, sulfamethoxazole and lamotrigine, and the number of dead cases were 280, 104, 87, 84 and 84, respectively (**Table 1**). The top five drugs analyzed by PRR most likely to induce DRESS were oxypurinol (PRR=745.31), aurotioprol (PRR=638.96), naproxen (PRR=212.99), iomeprol (PRR=199.96) and iobitridol (PRR=195.10) (**Table 2**). The top five drugs analyzed by PRR most likely to induce DRESS-related death was almotriptan, iobitridol, prasterone, canrenone and rifater (the combination of rifampicin, pyrazinamide and isoniazid), with the PRR of 736.38, 450.41, 320.16, 270.14 and 165.944 respectively (**Table 2**).

**Table 2:** Association analysis of DRESS and suspected drugs, sorted by proportional reporting ratio (PRR).

Drugs	Cases (n_1_)	PRR	χ^2^	Drugs with fatal outcome	Deaths (n_2_)	PRR	χ^2^
Oxypurinol	3	745.31	1549.52	Almotriptan	7	736.38	4406.07
Aurotioprol	6	638.96	3213.51	Iobitridol	6	450.41	2248.98
Naproxen	6	212.99	1063.85	Prasterone	7	320.16	1908.37
Iomeprol	87	199.96	16953.51	Canrenone	11	270.14	2660.83
Iobitridol	41	195.10	7714.77	Combination of rifampicin, pyrazinamide and isoniazid (Rifater)	6	165.94	821.67
Influenza vaccines	11	139.03	1373.38	Minocycline	74	126.80	8471.92
Ethacrynic acid	38	117.29	4261.26	Chlormezanone	3	125.75	256.51
Lysozyme	3	101.63	207.00	Siltuximab	4	99.90	298.11
Sertaconazole	3	89.44	181.57	Iopamidol	11	91.46	887.18
Sultamicillin	6	73.32	359.10	Trimebutine	8	87.74	597.98
Oxacillin	45	73.26	3130.79	Clemastine	8	77.99	529.89
Pyrazinamide	553	69.42	35962.85	Remifentanil	11	77.43	747.98
Cloxacillin	56	64.52	3431.34	Propylthiouracil	7	75.14	438.04
Cefotaxime	189	58.08	10561.15	Phloroglucinol	7	65.74	381.69
Kanamycin	27	58.59	1470.68	Dapsone	19	61.45	1051.65
Combination of rifampicin, pyrazinamide and isoniazid (Rifater)	6	165.94	821.67	Mianserin	18	56.11	904.91
Ethambutol	550	54.98	28116.87	Streptomycin	5	54.12	209.45
Rifampicin	1038	54.46	50904.99	Allopurinol	280	50.19	9847.91
Vancomycin	1564	47.95	64803.64	Oxacillin	3	45.56	89.81
Teicoplanin	125	45.77	5393.47	Phenobarbital	42	41.76	1566.11

### 3.2 Death of DRESS reviewed in case reports

**Figure 1:**
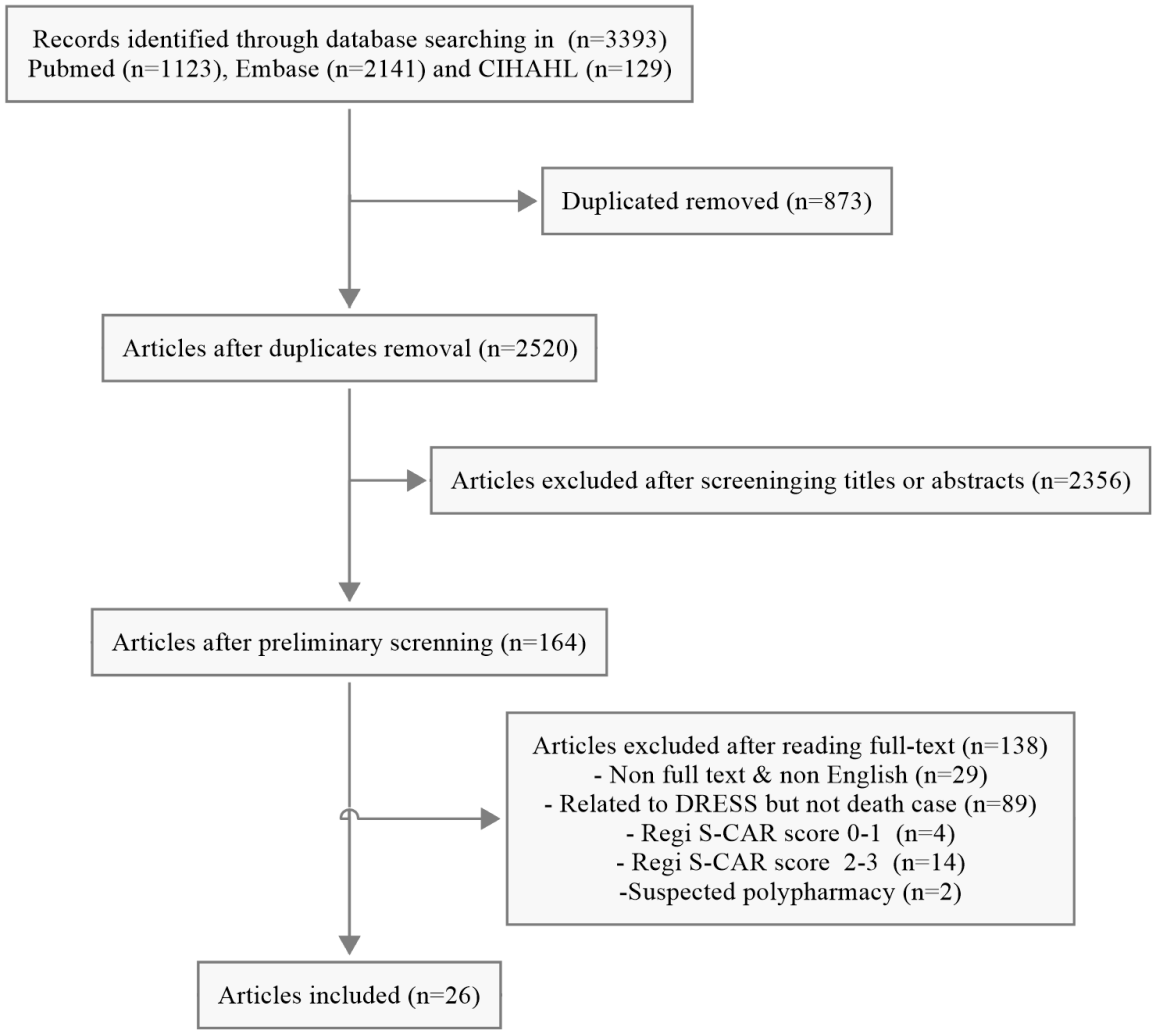
The detailed flow chart of the literature search.

A total of 3393 unique articles were initially retrieved. According to the inclusion and exclusion criteria, 26 individual cases from 26 publications were pooled for further analysis after screening (**Figure 1**).

### 3.3 Demographics and comorbidities

The age of patients in our systematic review ranged from 12–84 years (mean 55.13 years) for men and 1–86 years for women (mean 42.36 years). The number of male patients is slightly higher than that of female patients. The reported races included Caucasian, African, Asian, Turkish, Biracial, et al. In terms of comorbidities, there were relatively more patients combine with cardiac (7/26, 26.92%), endocrine and metabolic diseases (7/26, 26.92%).

### 3.4 Latency and reasons for hospitalization

DRESS patients characteristically experience a rash or fever before admission. The average time from dose to rash onset was 27.19 days (except for 4 patients not mentioned the exact time). Skin manifestations remained the main reason for admission (24/26, 92.31%), and the next was whole body issues (21/26, 80.77%).

### 3.5 Outcome

The majority of patients died in the hospital (23/26, 88.46%). Multi-organ problems were the most frequent cause of death (8/26, 30.77%), followed by respiratory (7/26, 26.92%), renal and liver (7/26, 26.92%). A complete list of the place of death and cause of death were presented in **Table 3**.

**Table 3:** Demographics, comorbidities, reasons for hospitalization, outcomes of the patients.

Gender	No. of cases	Age range (years)	Mean age (years)
Male	15 (57.69%)	12-84	55.13
Female	11 (42.31%)	1-86	42.36
Total	26	1-86	49.73
Race			
Caucasian	4 (15.38%)	Caucasian	
African	2 (7.69%)	African	
Asian	1 (3.85%)	Asian	
Turkish	1 (3.85%)	Turkish	
Biracial	1 (3.85%)	Biracial	
Not reported	17 (66.38%)	Not reported	
Comorbidities			
Cardiac	7 (26.92%)		
Endocrine and metabolic	7 (26.92%)		
Renal and liver	6 (23.08%)		
Infection	6 (23.08%)		
Skin	5 (19.23%)		
Whole body	5 (19.23%)		
Respiratory	3 (11.54%)		
Immune	3 (11.54%)		
Brain	2 (7.69%)		
Blood	2 (7.69%)		
Bone	2 (7.69%)		
Nervous	1 (3.85%)		
Gastrointestinal	1 (3.85%)		
Urinary	1 (3.85%)		
Reasons for hospitalization			
Skin	24 (92.31%)		
Whole body	21 (80.77%)		
Gastrointestinal	5 (19.23%)		
Respiratory	4 (15.38%)		
Immune	2 (7.69%)		
Urinary	2 (7.69%)		
Mental	1 (3.85%)		
Brain	1 (3.85%)		
Blood	1 (3.85%)		
Eye	1 (3.85%)		
Cardiac	1 (3.85%)		
Renal and liver	1 (3.85%)		
Not reported	1 (3.85%)		
Places of death			
In hospital	23 (88.46%)		
Not in hospital	3 (11.54%)		
Direct causes of death			
Multi-organ	8 (30.77%)		
Respiratory	7 (26.92%)		
Renal and liver	7 (26.92%)		
Infection	6 (23.08%)		
Cardiac	6 (23.08%)		
Brain	1 (3.85%)		
Skin	1 (3.85%)		
Time from the onset of the rash to the death			
≤30 days	12 (46.15%)		
30<t<100 days	6 (23.08%)		
≥100 days	5 (19.23%)		
Not mentioned	3 (11.54%)		
Organ involvement	Involved		
Skin	25 (96.15%)		
Liver	20 (76.92%)		
Pulmonary	9 (34.62%)		
Kidney	8 (30.77%)		
Cardiac	7 (26.92%)		
Pancreatic	1 (3.85%)		
Others	4 (15.38%)		
Laboratory indicators	Yes	No	N/A
Temperature (>38.5°C)	17 (65.38%)	4 (15.38%)	5 (19.23%)
Lymphomegaly	13 (50.00%)	2 (7.69%)	11 (42.31%)
	Average value	Range	Number of patients
Leukocytes (*10e9/L)	861.35 (8 N/A)	≤10	5 (19.23%)
		10~30	9 (34.62%)
		≥30	5 (19.23%)
		N/A	7 (26.92%)
Eosinophile granulocytes(*10e9/L)	111.51 (5 N/A)	≤0.5	2 (7.69%)
		0.5~1.5	4 (15.38%)
		≥1.5	15 (57.69%)
		N/A	5 (19.23%)
Alanine aminotransferase (ALT, IU/L)	594.37 (11 N/A)	≤40	1 (3.85%)
		40~120	6 (23.08%)
		≥120	9 (34.62%)
		N/A	10 (38.46%)
Aspartate aminotransferase (AST, IU/L)	1650.83 (11 N/A)	≤40	1 (3.85%)
		40~120	6 (23.08%)
		≥120	8 (30.77%)
		N/A	10 (38.46%)
Alkaline phosphatase (ALP, IU/L)	466.05 (18 N/A)	≤150	0
		150~450	5 (19.23%)
		≥450	4 (15.38%)
		N/A	17 (65.38%)
Direct bilirubin (DBil, mg/dl)	5.52 (20 N/A)	≤0.25	0
		0.25~0.75	0
		≥0.75	7 (26.92%)
		N/A	19 (73.08%)
Total bilirubin (TBil, mg/dl)	9.61 (18 N/A)	≤1	0
		1~3	1 (3.85%)
		≥3	8 (30.77%)
		N/A	17 (65.38%)

### 3.6 Organ involvement

Multi-organ involvement was common (16/26, 61.54%). The most frequently affected visceral organs being the skin (25/26, 96.15%), while liver involvement occurred in (20/26, 76.92%). Pulmonary (9/26, 34.62%), kidney (8/26, 30.77%), cardiac (7/26, 26.92%) involvement was relatively common. Pancreatic occurred in only 1 out of 26 patients (3.85%). Others included esophagus, stomach, duodenum, sigmoid colon, digestive, eyes, colon, re-epithelialized mucosa of the descending colon, sigmoid colon, and rectum, were less common.

### 3.7 Laboratory indicators

In our systematic review, 18 of 27 patients (66.67%) presents fever (>38.5°C), while almost half patients showed lymphomegaly (48.15%). The leukocyte count was 861.35*10e9/L, ranging from 0.0121 to 16,000 cells/L. The eosinophile granulocyte count was 111.51*10e9/L, ranging from 0 to 2240 cells/L. One patient had normal ALT and AST, while 9 (34.62%), 8 (30.77%) and 4 (15.38%) patients had ALT, AST and ALP three times higher than normal, respectively. Seven patients with abnormal DBil values had DBil values more than three times normal. As for Tbil, 30% of patients had Tbil values three times greater than normal.

### 3.8 Putative causative agents

**Figure 2:**
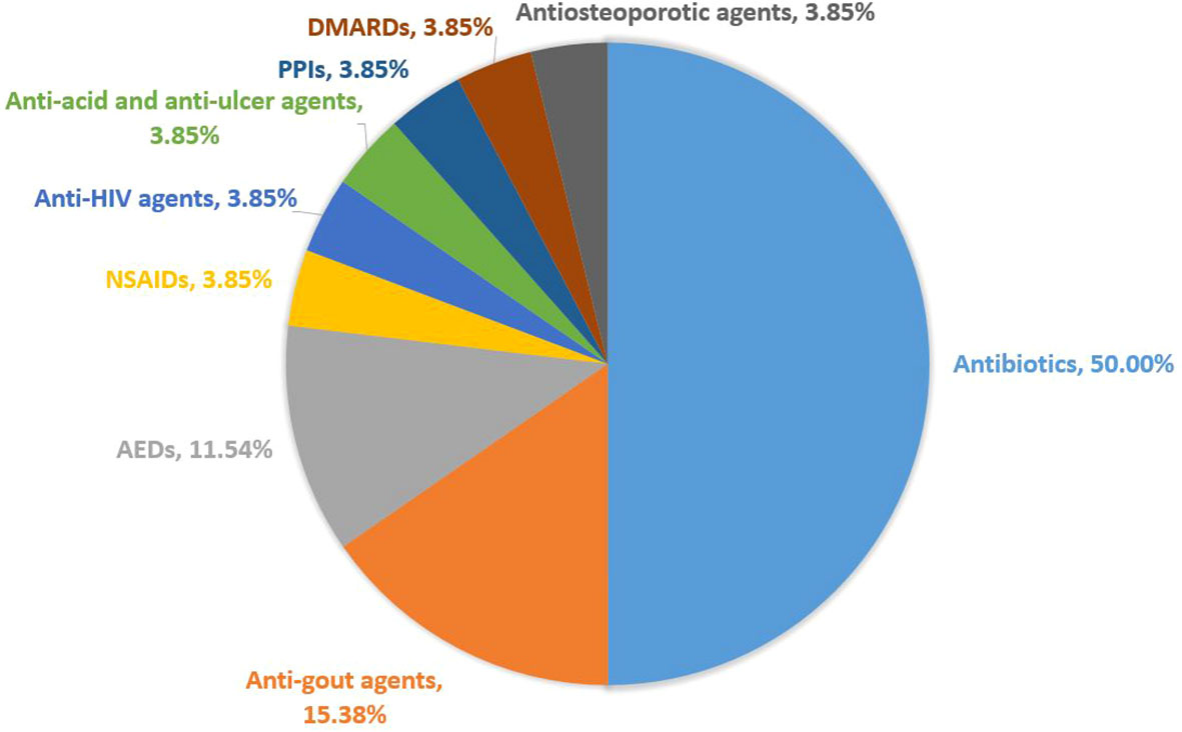
Causative drugs related to fatal outcome of DRESS cases.

As shown in **Figure 2** and **Table 4**, the most commonly suspected medications were antibiotics (13/26, 50.00%), followed by anti-gout agents (4/26, 15.38%) and anti-epileptic drugs (AEDs) (3/26, 11.54%). Others including NSAIDs, anti-HIV agents, anti-acid and antiulcer drugs, proton pump inhibitors (PPIs), disease-modifying anti-rheumatic drugs (DMARDs) and antiosteoporotic agents, only reported one case respectively (1/26, 3.85%). More details of the case reports included in the systematic review are shown in **Table 5**.

**Table 4:** List of the culprit medication in patients with DRESS syndrome ended in death.

Culprit Medication	Number of cases
Allopurinol	4 (15.38%)
Minocycline	3 (11.54%)
Vancomycin	2 (7.69%)
Amoxicillin and clavulanate, azithromycin, clindamycin, colistimethate, dolutegravir, lansoprazole, leflunomide, levetiracetam, nabumetone, omeprazole, oxacillin, phenytoin, pyrazinamide, strontium ranelate, sulfasalazine, sultamicillin, zonisamide	17 (65.38%)
Total	26

**Table 5:** Details of DRESS related death reported in case reports and case series.

No.	Articles	Age	Gender	Medicine	Comorbidities	Cause of death	RegiSCAR criteria
1	Yaylacı, S., 2012 (4)	70	M	Allopurinol	Gout	Acute renal failure and acute liver failure	7
2	Choi, H. G., 2014 (5)	84	M	Allopurinol	Stage 3 chronic renal disease	Sudden cardiac arrest	4
3	Uppalapati, A., 2014 (6)	40	F	Lansoprazole	Liver cirrhosis	Multiorgan failure	5
4	Sato, H., 2022 (7)	59	M	Levetiracetam	Aphasia	Multiple organ system failure secondary to acute fulminant liver injury from atypical DIHS	6
5	Desai, A. S., 2022 (8)	76	M	Oxacillin	New-onset heart failure in the context of S. Aureus bacteremia	Extensive pneumonia	6
6	Boumitri, C., 2017 (9)	64	M	Vancomycin	Infected right hip prosthesis	Cytomegalovirus infection	>5
7	Martin, C., 2018 (10)	59	M	Dolutegravir	HIV-positive, pulmonary tuberculosis	Liver decompensation	9
8	Sibille, L., 2017 (11)	72	M	Nabumetone	Gastrointestinal and bone issues	Lung infiltrate	6
9	Kombila, U. D., 2018 (12)	35	M	Pyrazinamide	First episode of a smear positive pulmonary tuberculosis	Liver failure	4
10	Gude, D., 2011 (13)	86	F	Phenytoin	Stroke,seizure disorder	Severe sepsis, respiratory failure, cardiac arrest	4
11	Montecchiani, V., 2017 (14)	40	F	Allopurinolinduzierte	Active pulmonary tuberculosis (TB)	Multi-organ failure	6
12	He, Q., 2020 (15)	66	F	Omeprazole	Abdominal distention	Renal failure	9
13	Drago, F., 2016 (16)	71	F	Strontium ranelate	Osteoporosis	Cardiac and respiratory insufficiencies	7
14	Plost, G., 2017 (17)	12	M	Minocycline	Folliculitis	Complications of multisystem organ failure	6
15	Miller Quidley, A., 2012 (18)	63	F	Clindamycin	Methicillin-susceptible Staphylococcus aureus (MSSA) prosthetic hip joint infection	Life-sustaining measures were withdrawn at the family’s request	9
16	Micozzi, S., 2015 (19)	20	F	Minocycline	Acne	Primary graft failure	6
17	Pursnani, A., 2009 (20)	48	M	Azithromycin	Upper respiratory tract infection	Sepsis	5
18	Numéro, N. J. R. A., 2011 (21)	19	F	Minocycline	Acne	Heart transplantation	4
19	Ardérius, M., 2018 (22)	28	F	Sulfasalazine	Psoriatic arthritis	Cardiogenic shock and multiorgan failure;anoxic encephalopathy	6
20	Coban-Karatas, M., 2012 (23)	31	M	Amoxicillin and clavulanate	Flu	Progressive liver damage and fulminant hepatitis	6
21	Karimpour, H., 2020 (24)	28	M	Colistimethate sodium, phenytoin, meropenem, quetiapine, levofloxacin, vancomycin	Decreased levels of consciousness	Septic shock	5
22	Kitcharoensakkul, M., 2012 (25)	1.8	F	Vancomycin	Methicillin-resistant Staphylococcus aureus pericarditis with bacteremia status post subtotal pericardiectomy	Respiratory and cardiac arrest	6
23	Kato, M., 2016 (26)	68	M	Allopurinol	Hyperuricaemia	Septic shock	7
24	Takamiyagi, S., 2022 (27)	66	M	Zonisamide	Numbness after thalamic hemorrhage	Respiratory failure caused by pneumonia	7
25	Chen, H. C., 2022 (28)	55	M	Sultamicillin	Eosinophilia and Systemic Symptoms Syndrome Patients With Lymph Node	Septic shock	6
26	Fatima, M., 2023 (29)	32	F	Leflunomide	Joint pains	Worsening skin lesions	7

## 4 Discussion

DRESS is a life-threatening multi-organ system reaction. DRESS was often overlooked in clinical practice due to its unique clinical presentation, such as delayed onset and similarity to symptoms of other conditions. DRESS has a heterogenous clinical presentation making it challenging to diagnose, typically developed by assessing the timing of drug exposure in relation to the onset of skin rash and the resolution of symptoms following discontinuation of the offending drug. Recently, Brüggen et al. (30), reported a delphi-based international consensus on diagnostic workup, severity assessment, and management of DRESS, but it did not cover all aspects of DRESS management in detail and some topics remained controversial. Therefore, the development of more researches and future guidelines are necessary. The main advantages of our research is that we integrated the FAERS database analysis with case report assessments and a systematic review, effectively reducing the risk of bias that might come from using just one single database.

Results showed that allopurinol has the highest probability to induce DRESS and related death statistically. A comprehensive analysis (31) including 52 case reports revealed a mortality rate of 25.0% of allopurinol-induced DRESS, with septic shock, gastrointestinal bleeding, and multiple organ failure identified as the primary causes of death. Factors associated with increased risk of mortality included advanced age, underlying cardiovascular disease, chronic kidney disease, high allopurinol dosage, infection, and internal organ involvement such as the kidney, heart, lung, and gastrointestinal tract. In our systematic review, two patients (50%) had chronic renal failure, that may be related to their poor prognosis. The most common visceral manifestation were liver and kidney injuries. The human leukocyte antigen (HLA) class I alleles HLA-B*58:01 and HLA-C*03:02 have been implicated in allopurinol-induced SCAR, with HLA-C*03:02 being predominantly associated with DRESS (32). These findings underscore the importance of pre-treatment HLA genotyping in patients with compromised renal function to mitigate the risk of DRESS. Chronic renal failure was the cause of 2 out of 4 deaths caused by allopurinol in our systematic review. HLA associations with DRESS have provided insights into immunopathogenesis. In some SCAR cases, HLA oligoclonal CD8+ T-cell responses restricted by HLA are observed at the tissue level (33). Nevertheless, for the majority of drugs, the specific HLA risk alleles and antigens that drive this response remain unidentified. Additionally, HLA risk alleles exhibit incomplete positive and negative predictive values, rendering comprehensive screening a current challenge. Recently, single-cell RNA sequencing has been proved helpful in guiding therapeutic decisions on DRESS patients (34).

A systematic review (35) identified 1072 cases of psychotropic drug-induced DRESS indicated that carbamazepine, lamotrigine, phenytoin, valproate, and phenobarbital were the most implicated drugs. Studies have examined the genetic contributions to cutaneous adverse drug reactions caused by AEDs and discovered significant associations with HLA molecule genes, the cytochrome P450 enzyme and complement factor H genes (36). One case (1/26, 3.85%) in our systematic review were related to phenytoin, but the patient did not do any genetic test. Significant genetic factors have been identified in association with phenytoin-related SCAR. Patients with SCAR, particularly those carrying the CYP2C9*3 allele, exhibited delayed plasma phenytoin clearance (37). The alleles HLA-B*13:01, HLA-B*15:02, and HLA-B*51:01 demonstrated significant associations with phenytoin hypersensitivity, each exhibiting distinct phenotypic specificities (38). Therefore, integrating the evaluation of HLA and CYP2C9 risk alleles enhances the efficacy of predictive genetic testing for the prevention of phenytoin hypersensitivity in Asians. Although there were no deaths caused by lamotrigine among the deaths included in this article, lamotrigine ranked second in drugs calculated to induce DRESS and fifth statistically related to fatal outcome. In Asian populations, HLA-B*1502 increases the risk of lamotrigine- induced bullous lesions, while HLA-A*2402 is linked to susceptibility to SCARs (39). Conversely, HLA-A*3303 serves as a protective allele against lamotrigine- induced SCARs (39).

A review conducted in the USA (1980 to 2016) found that antibiotics, particularly vancomycin (39%), β-lactams (23%), fluoroquinolones (4%), tetracyclines (4%), and sulfonamides (3%), were implicated in total 74% of DRESS syndrome cases (40). In our study, vancomycin ranked third among the drugs related to DRESS and ranked second among the drugs statistically related to death. Vancomycin related DRESS syndrome happened within 21 days (41). A systematic case review (42) which analysis the immune-mediated reactions to vancomycin indicated that the incidence of DRESS syndrome was 16/71, 23%, men represented a majority (56%) with median latency of 21 days [IQR 17 days, 28 days] for DRESS syndrome. Our results showed that the time from the onset of the rash to the death with vancomycin were 40 to 56 days, which means once rash begins to appear, the next 20-30 days are very valuable for treatment. It is noteworthy that other glycopeptide antibiotics are also possible to cause DRESS syndrome in limited number of patients and may lead to allergic cross-reactivity with vancomycin (43). Vancomycin-associated DRESS may be due to genetic factors. A study identified that 20% of HLA-A*32:01 patients would develop vancomycin DRESS if exposed for more than two weeks (44). Another study reported the cross-reactivity of alternative glycopeptide antibiotics, namely teicoplanin and telavancin, in patients exhibiting HLA-A*32:01–restricted vancomycin-induced DRESS, who also share the expression of an HLA class II haplotype, specifically DQA1*01:01 and DQB1*05:03 (45). A PCR-based assay specific to HLA-A*32:01 has been developed to screen patients for the risk of vancomycin-induced DRESS (46).

Among the deaths included in this article, 3 patients were related to DRESS of minocycline, and 2 of them had cardiac involvement. Dyspnea and chest pain are the most prevalent symptoms in patients with DRESS and cardiac involvement, with minocycline being identified as one of the most frequently implicated medications (47). Due to the high mortality rate of DRESS with cardiac involvement (45%) (47), doctors and pharmacists should pay special attention to the chest pain or abnormal heartbeats of patients. In the systematic review, the cardiac damage lead to death in DRESS cases included sudden cardiac arrest, heart transplantation after giant cell myocarditis (GCM), cardiac insufficiencies and cardiogenic shock. When dealing with DRESS patients, ECG, echocardiogram, cardiac enzymes and troponin should be performed, If necessary, perform endomyocardial biopsy as well. Physicians should pay attention to the occurrence of chest pain, sinusoidal, ischemic, depressed injection fraction, giant cell myopathy, and fulminant eosinophilic necrotizing myopathy, which may be associated with a higher possibility of death.

The antibiotics mentioned in this study include minocycline (3/13, 23%), vancomycin (2/13, 15%), amoxicillin and clavulanate, azithromycin, clindamycin, colistimethate, oxacillin, pyrazinamide, sulfasalazine and sultamicillin (1/13, 0.69% each). Previous research (41) has shown that the severity of antibiotic-induced DRESS syndrome compared to other triggers remains a topic of debate, but our research suggests that antibiotic related DRESS has a relatively high mortality rate (50%). Therefore, clinicians should exercise caution when prescribing antibiotics to patients with a history of DRESS syndrome.

So far, rash remained the most frequent clinical findings. The skin disorders in fatal cases were mostly severe, systemic and diffuse, with features that included rash, erythema, maculopapulars, urticarial rash, pustules. The generalized rash could be with or without itching, edema, esquamation, blisters, scabs, erosions. The rash could appear on the trunk, extremities and face, and could started from one part of the body and spread to the entire body. Out of 26 cases, 10 have undergone skin biopsy and all were consistent with DRESS. Six skin biopsy (6/10, 60%) mentioned dermatitis or perivascular inflammatory infiltrate with elevated eosinophils. One skin lesion (1/10, 10%) showed liquefaction degeneration in the epidermis. One skin biopsy (110, 10%) for direct and indirect immunofluorescence revealed linear C3 deposits and IgG deposits, respectively. ELISA testing for BP 180 and 230 was positive, consistent with a diagnosis of bullous pemphigoid. A study retrospective study on 50 skin biopsies from 36 patients with DRESS syndrome, and demonstrated that 28% with typical lymphocytes (most with CD8 expressed) and 6% with T-cell (48). Different types of SCARs may differ in terms of the specific effector T cells involved and the chemokines/cytokines released, resulting in cell homing to skin and specific tissue targeting (33).

Virus reactivation were observed in 4 fatal cases in the systematic review, of which 2 cases referred to HHV reactivation (including HHV-6 and HHV-7), and 2 cases mentioned cytomegalovirus (CMV) reactivation. In addition, there were 5 cases reported as virus infection. A study prospectively assessed 40 patients exhibiting DRESS found that EBV, HHV-6 or HHV-7 reactivation in 76% of the patients (49). In our systematic review, this proportion was lower, that possibly due to two reasons. On the one hand, some cases did not undergo virus testing, and on the other hand, some cases that tested positive for the virus were not determined whether they belonged to reactivation. Virus reactivation is related to the onset of DRESS, because the viral reactivation in DRESS cases is thought to lead to the circulating CD8+ T lymphocytes activation (49). HHV reactivation is potentially linked to increased severity of reactions, with viral reactivation correlating to the level of inflammation (50). A reactivation can be asymptomatic, or it may cause prolonged symptoms in DRESS, long after stopping the causative drugs (50).

Among the drugs mentioned in this article, allopurinol, minocycline, vancomycin, amoxicillin and clavulanate, leflunomide, levetiracetam, nabumetone, sulfasalazine, zonisamide and azithromycin have warning signs for DRESS in their drug instructions, while colistimethate, dolutegravir, lansoprazole, omeprazole, oxacillin, phenoin, pyrazinamide do not have warning signs in their drug instructions. It is necessary to remind patients in the drug labels that serious skin reactions, including dress may develop, and advise patients to stop taking suspect drug immediately if they develop any type of rash or fever. The patients should discontinue the suspected drug immediately if symptoms occur and contact their healthcare provider as soon as possible.

The initial step in managing DRESS involves discontinuing the suspected drug responsible. Systemic corticosteroids continue to be the preferred treatment option. Systemic corticosteroids continue to be the preferred treatment option. However, several retrospective studies indicated that systemic corticosteroids associated with higher risk of DRESS relapse (51), viral reactivation (51, 52), various infections (53), and development of autoimmune diseases (53). Although the role of steroid-sparing therapies in DRESS is still not well-established yet, there is increasing evidence supporting the use of steroid-sparing therapies such as cyclosporine, intravenous immunoglobulin (IVIG), interleukin (IL)-5 axis inhibitors, and Janus kinase (JAK) inhibitors (54). Although biomarkers not measured in the cases reviewed in this article, in light of the ongoing diagnostic challenges associated with DRESS, there is a growing interest in investigating biomarkers. Ogawa’s study revealed a significant increase in serum thymus and activation-regulated chemokine (TARC/CCL17) levels among patients with DRESS compared to those with morbilliform drug eruption and Stevens-Johnson syndrome/Toxic epidermal necrolysis (SJS/TEN) (55). In fact, a prospective case control study demonstrated comparable efficacy between serum TARC levels and a RegiSCAR score (≥2) in distinguishing cases of DRESS from controls with maculopapular drug rash (56). Miyagawa reported that Th2-associated chemokines were markedly upregulated in DIHS/DRESS (57). IFN-γ ELISpot assay (58, 59) and IFN-γ combined with IL-4/IL-2 (56, 60) could offer potential for use as rapid diagnostic tests.

It must be admitted that the study encountered various limitations. Firstly, the FAERS data utilized in our analysis were subject to voluntary reporting by healthcare professionals and consumers, potentially resulting in variations in report quality due to the skills and diligence of the reporters. This could introduce bias into our findings through missing, inadequate, or incomplete reports. Additionally, FAERS lacks comprehensive information on patient morbidity related to drug use, as it does not provide data on the total number of patients using the drug. Furthermore, the small sample size of case reports limited our ability to do risk factor analysis. Lastly, methodological constraints prevented us from establishing a causal relationship between drug and the observed positive signals or reported cases.

## 5 Conclusion

To our knowledge, this article is the first study that focus on fatal DRESS cases. Our research indicates a relatively high mortality rate associated with antibiotic-related DRESS. Cardiac involvement is the most common comorbidities among DRESS deaths, and patients taking high-risk medication should be carefully monitoring.

## Data Availability

The original contributions presented in the study are included in the article/supplementary material. Further inquiries can be directed to the corresponding author.
